# Microbubbles detection during cardiopulmonary bypass with transoesophageal echocardiography: a case report

**DOI:** 10.1186/1757-1626-1-141

**Published:** 2008-09-05

**Authors:** Paolo Zanatta, Enrico Bosco, Valeria Salandin, Loris Salvador, Carlo Valfrè, Carlo Sorbara

**Affiliations:** 1 Anesthesia and Intensive Care Department, Treviso Regional Hospital, Piazzale Ospedale n°1, 31100 Treviso, Italy; 2 Cardiovascular Desease Departement, Treviso Regional Hospital, Piazzale Ospedale n°1, 31100 Treviso, Italy

## Abstract

**Introduction:**

Microembolic signals are usually detected with transcranial doppler during cardiac surgery.

This report focuses on suggesting the transesophageal echocardiography as a different diagnostic approach to detect microemboli during cardiopulmonary bypass.

**Case presentation:**

A 58 year old male patient, caucasian race, was operated on video assisted minimally invasive mitral valve repair using right minithoracotomy approach. His past medical history included an uncontrolled hypertension, dyslipidemia, insulin dependent diabetes mellitus, carotid arteries stenosis. The extracorporeal circulation was performed with femoral-femoral artery and venous approach. Negative pressure for vacuum assist venous drainage was applied in order to facilitate venous blood return. The patient had a brain monitoring with bilateral transcranial doppler of middle cerebral arteries and a double channels electroencephalogram. A three dimensional transesophageal echocardiography to evaluate the mitral valve repair was performed.

During the cardiopulmonary bypass a significant microembolic activity was detected in the middle cerebral arteries spectrum velocities due to gas embolism from venous return. Simultaneous recording of microbubbles was also observed on the descending thoracic aorta transesophageal echo views.

**Conclusion:**

During the aortic cross-clamping time the transesophageal echocardiography can be useful as an alternative method to assess the amount of gas embolism coming from cardiopulmonary bypass. These informations can promote immediate interaction between perfusionist, surgeon and anesthesiologist to perform adequate manoeuvres in order to reduce the microembolism during extracorporeal circulation.

## Introduction

The microembolic injury is one of the determining factors to cognitive dysfunction after cardiac surgery [1] as hypoperfusion, iper-rewarming and inflammation due to cardiopulmonary bypass (CPB) [2].

In literature the transcranial doppler (TCD) is proposed to determine the occurrence and the frequency of cerebral microembolic signals during different kinds of cardiac surgery and may alert the surgical team when microemboli enter into the cerebral circulation during surgery, thus allowing preventive measures to be taken [3].

Advances in doppler technology have made possible to detect not only gaseous microemboli but also the solid ones [4], derived from pericardial blood suction [5] and platelets aggregation on gas microbubbles [6].

Microbubbles are normally seen with transoesophageal echocardiography (TEE) in the heart after declamping the aorta especially in patients submitted to valve surgery. Normally the TEE evaluation stops when CPB starts and the surgeon clamps the aorta: at this time it is possible to explore the descending thoracic aorta long and short axis with TEE. We suggest to utilize these echo windows to detect microbubbles coming from the extracorporeal circulation.

We have documented this case because it describes an interesting example of TCD and TEE interaction.

## Case presentation

A 58 year old male patient with severe mitral valve insufficiency was scheduled for a minimally invasive mitral valve surgery (MIMVS) repair which consists of a video assist right minithoracotomy.

His past medical history included uncontrolled hypertension, dyslipidemia, insulin dependent diabetes mellitus, epiaortic vessel stenosis (50% stenosis in the left and in the right internal carotid arteries) and a recent acute heart failure.

The preoperative echocardiography revealed a mitral valve insufficiency because of a posterior leaflet prolapse with a moderate reduction of the ejection fraction.

The three dimensional TEE helped to assess the function of mitral valve before and after surgical repair.

Bilateral middle cerebral arteries velocity, emboli count and differentiation were recorded by TCD (Doppler Box – DWL). A longitudinal bipolar electroencephalogram montage (2 channels: F3-C3 and F4-C4)) was used, based on the International 10–20 System. The neurophysiological monitoring system (Eclipse – Axon System) simultaneously recorded the raw, the spectral EEG and two videos from TCD and from TEE.

A moderate hypothermic (34°C) phosporilcoline coated circuit (Dideco Avant) with venous (25 Fr Cardiovation) and arterial (22 Fr DLP) femoral cannulations was performed. A vacuum assist venous drainage of at least 60 mmHg was applied to the external reservoir system to facilitate the venous return according to the surgeon demand. The circuit had a bubble trap of 40 μm on the arterial line and a roller pump (Stokert SV). The aorta was clamped by the surgeon and the Custodiol cardioplegic solution was perfused on the ascending aorta as a single shot. CO_2 _was continuously delivered at 3 to 5 l/min into operative field until the left atrium was de-aired and closed.

## Results

**Figure 1:**
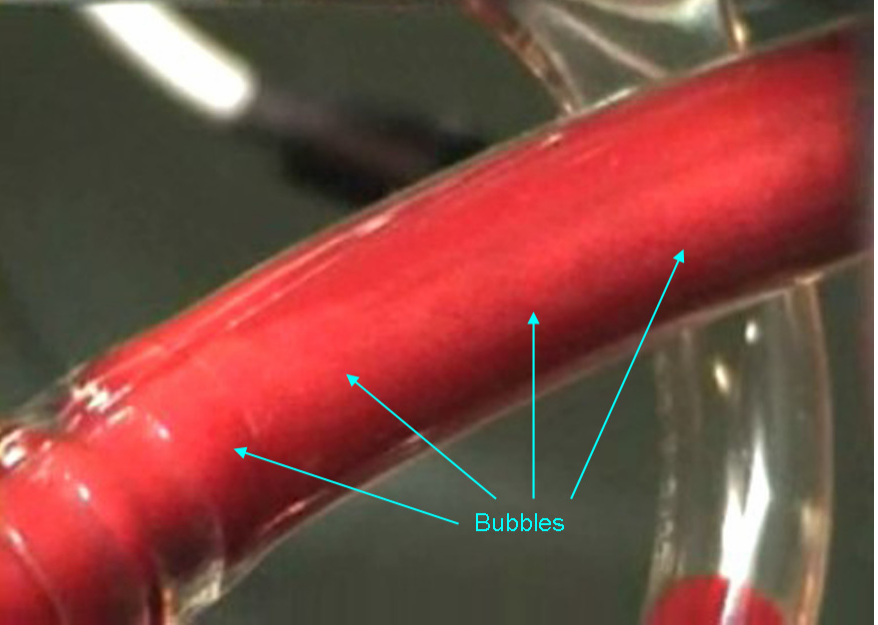
Gas bubbles like foam in the venous return line.

**Figure 2:**
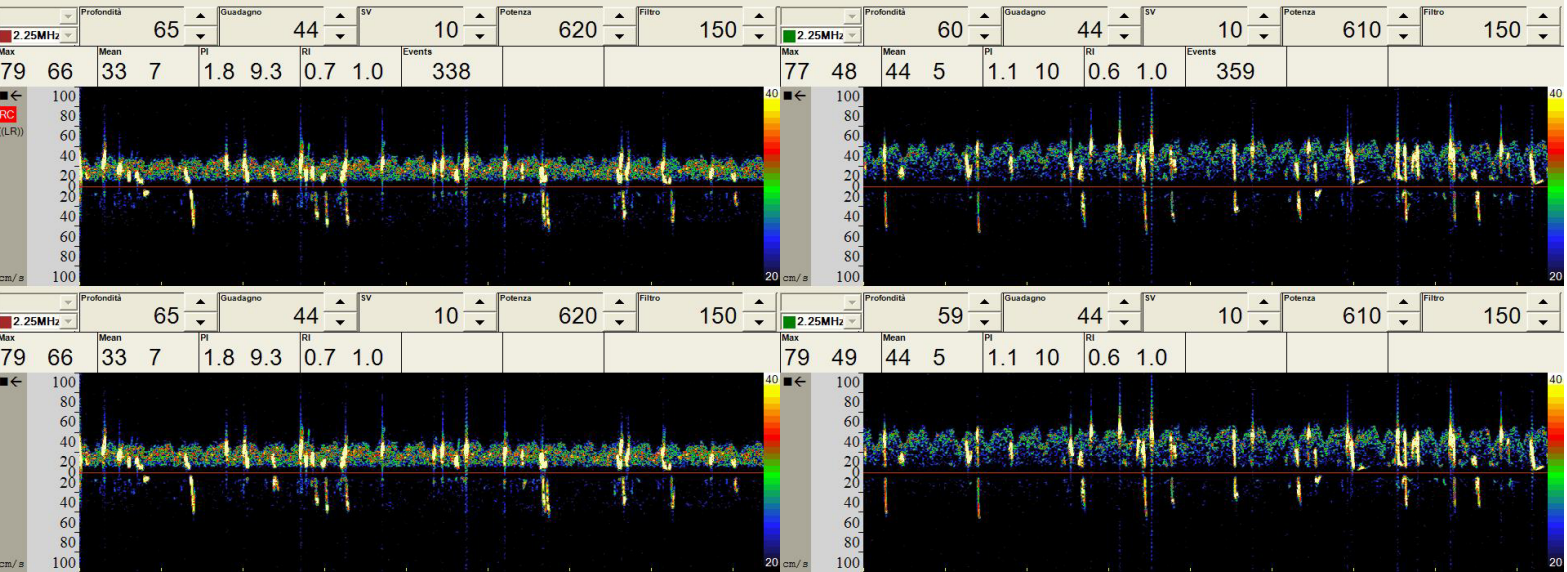
MES from TCD obtained with double sampling boxes placed over the two middle cerebral arteries.

**Figure 3:**
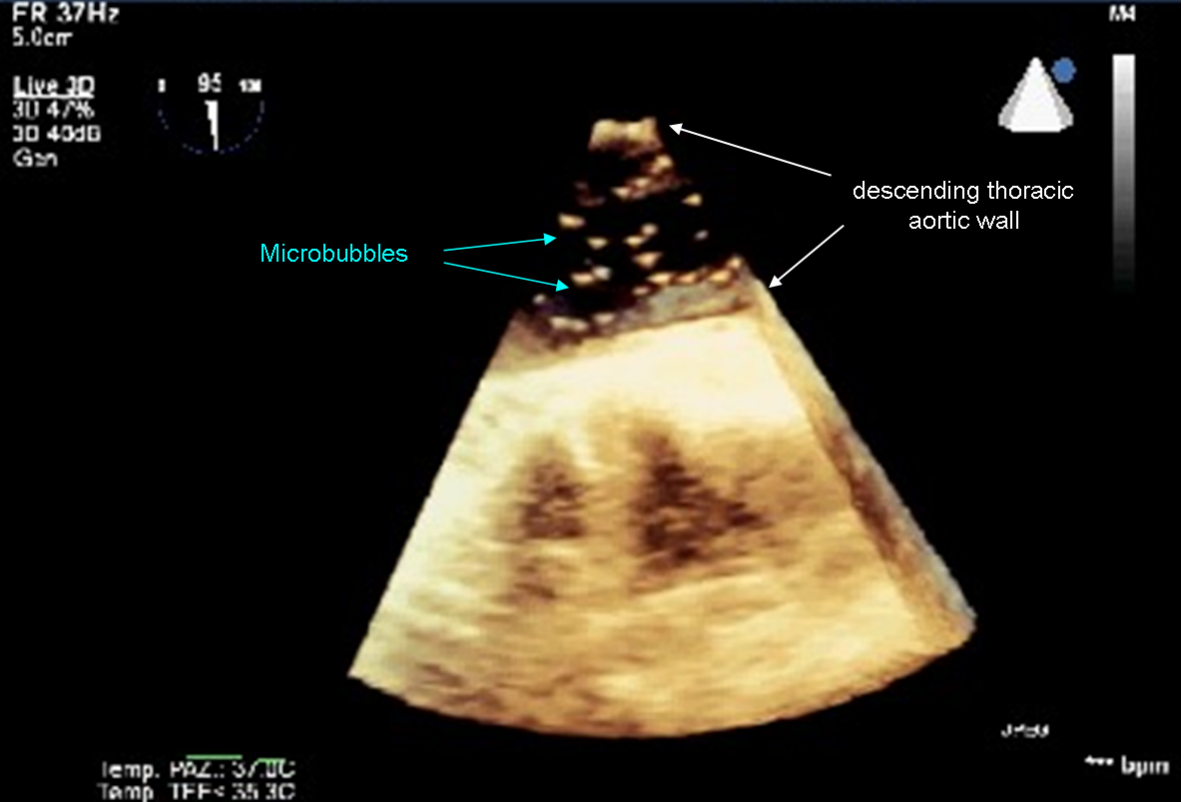
Microbubbles recorded from descending thoracic aorta three dimensional TEE.

No significant microembolic activity was recorded until the extracorporeal circulation started. After the aorta was clamped and cardioplegic perfusion made, the surgeon opened and mechanically fixed the left atrial wall to visualize the mitral valve. During this time the TCD revealed bilateral microembolic signals reaching the brain. During six minutes of monitoring the TCD software recorded 213 MES on the left middle cerebral artery (178 gaseous and 35 solid) and 234 MES on the right one (220 gaseous and 49 solid) (Tab 1). The perfusionist noted gas bubbles like foam on the venous return line (Fig 1). We appreciated MES on TCD (Fig 2) and gas microbubbles on TEE (Fig 3) and simultaneously these audio/video informations were recorded in the neurophysiological monitoring system [see Additional file 1].

**Table 1:** Gaseous and solid MES differentiation on the left and right meddle cerebral arteries.

	Left	Right	Total
Gasseous	178	220	398
Solid	35	14	49
Total	213	234	447

This microembolic activity disappeared after eight minutes; during this time the vacuum assist venous drainage was set below 40 mmHg.

## Discussion

Several studies have demonstrated the impact of microemboli during CPB on postoperative neurological dysfunction [1,7].

TCD is capable of detecting microembolic material, both gaseous and solid, within the intracranial cerebral arteries and his utility in determining the occurrence and the frequency of high intensity transient signals (HITS) during different cardiac surgery procedures has been documented [3].

TCD is operator dependent and requires training and experience neurologist to perform and to interpret results [8]. In the daily clinical practice most of the cardiac anaesthesiologists is able to perform a routine intraoperative TEE according to the recommendations of the American Society of Echocardiography published in the 1999 [9]. In the clinical practice the identification of gas microbubbles by TEE is limited to the dearing time before declamping the aorta and the outpatient test of patent forame ovale since the TCD and TEE showed an almost perfect concordance in detection and quantification of right-left shunt [10]. In both two different clinical scenarios the echo window utilized is the four chamber mid esophageal. So far nobody has proposed to identify HITS during CPB with TEE on descending thoracic aorta views.

**Figure 4:**
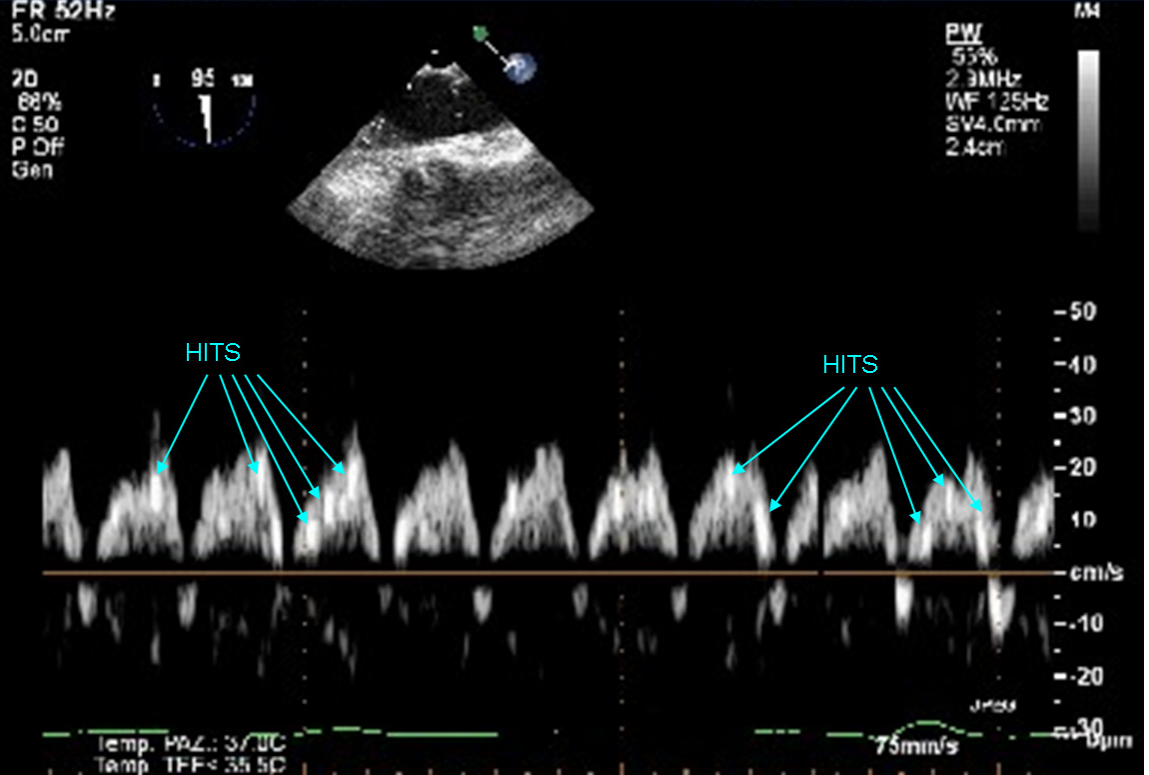
Two dimensional TEE Pulse Wave Doppler recording of HITS in the descending thoracic aorta long axis view during CPB.

The TEE could asses the systemic embolic load during the extracorporeal circulation. Moreover TEE could, like TCD, monitors the surgical, perfusionist and anesthesiologist procedures in respect to the air contamination of CPB. Like the TCD the Pulse Wave Doppler of the descending thoracic aorta blood flow can visualize the microbubbles as HITS because of a different intensity of the Doppler signal due to microemboli (Fig 4). The current TCD software with an automatic emboli detection and count is not implemented in the echocardiography devices.

In our patient a big amount of MES is recorded during the CPB when an higher negative pressure over 60 mmHg is necessary to achieve the venous drainage trough a 25 Fr trans-femoral cannula. In literature an high level of vacuum is reported to be a risk factor for gas embolism during the extracorporeal circulation [11]. A reduced emboli count during CPB is observed when the venous drainage is obtained with a double (femoral and jugular) venous cannulation and a reduced negative pressure is applied to the venous return [12].

## Conclusion

The TEE monitoring of the descending thoracic aorta during the CPB seems to be an alternative method in respect to TCD to assess the microembolic activity and could be a new approach to monitor the efficiency of the surgical team and of the bypass circuit regarding the systemic gas microembolization.

Our case seems to sustain higher microembolic load in patient in which minimally invasive mitral valve surgery is performed with one percutaneous venous cannula and high level of vacuum in the venous return.

## Abbreviations

TCD: Transcranial Doppler; EEG: Electroencephalogram; TEE: Transoesophageal Echocardiography; MES: Microembolic Signals; MIMVS: Minimally Invasive Mitral Valve Surgery; CPB: Cardiopulmonary Bypass.

## Competing interests

The authors declare that they have no competing interests.

## Authors' contributions

PZ conceived the work, carried out the study, collected and analyzed the data and wrote the article. EB, VS and LS analyzed the data and helped to write the article. CV and CS analysed the data. All authors read and approved the final manuscript.

## Consent

Written informed consent was obtained from the patient for publication of this case report and accompanying images. A copy of the written consent is available for review by the Editor-in-Chief of this journal.

## Supplementary Material

Additional file 1 Movie 1. Simultaneously recording of EEG, microbubbles with 3d TEE and MES with TCD during CPB. The audio is from the TCD: is it possible to distinguish the sound from MES and the one from blood flow because of the roller pump. Click here for file

## References

[B1] Pugsley W, Klinger L, Paschalis C, Treasure T, Harrison M, Newman S (1994). The impact of microemboli during cardiopulmonary bypass on neuropsychological functioning. Stroke.

[B2] Grocott HP, Homi HM, Puskas F (2005). Cognitive dysfunction after cardiac surgery: revisiting etiology. Semin Cardiothorac Vasc Anesth.

[B3] Brækken SK, Russell D, Brucher R, Abdelnoor M, Svennevig JL (1997). Cerebral Microembolic Signals During Cardiopulmonary Bypass Surgery Frequency, Time of Occurrence, and Association With Patient and Surgical Characteristics. Stroke.

[B4] Russell D, Brucher R (2002). Online automatic discrimination between solid and gaseous cerebral microemboli with the first multifrequency transcranial Doppler. Stroke.

[B5] Kincaid EH, Jones TJ, Stump DA, Brown WR, Moody DM, Deal DD, Hammon JW (2000). Processing scavenged blood with a cell saver reduces cerebral lipid microembolization. Ann Thorac Surg.

[B6] Barak M, Katz Y (2005). Microbubbles: pathophysiology and clinical implications. Chest.

[B7] Barbut D, Lo YW, Gold JP, Trifiletti RR, Yao FS, Hager DN, Hinton RB, Isom OW (1997). Impact of embolization during coronary artery bypass grafting on outcome and length of stay. Ann Thorac Surg.

[B8] Sloan MA, Alexandrov AV, Tegeler CH, Spencer MP, Caplan LR, Feldmann E, Wechsler LR, Newell DW, Gomez CR, Babikian VL, Lefkowitz D, Goldman RS, Armon C, Hsu CY, Goodin DS Therapeutics and Technology Assessment Subcommittee of the American Academy of Neurology. Assessment: transcranial Doppler ultrasonography: report of the Therapeutics and Technology Assessment Subcommittee of the American Academy of Neurology. Neurology.

[B9] Shanewise JS, Cheung AT, Aronson S, Stewart WJ, Weiss RL, Mark JB, Savage RM, Sears-Rogan P, Mathew JP, Quiñones MA, Cahalan MK, Savino JS (1999). ASE/SCA guidelines for performing a comprehensive intraoperative multiplane transesophageal echocardiography examination: recommendations of the American Society of Echocardiography Council for Intraoperative Echocardiography and the Society of Cardiovascular Anesthesiologists Task Force for Certification in Perioperative Transesophageal Echocardiography. Anesth Analg.

[B10] Belvís R, Leta RG, Martí-Fàbregas J, Cocho D, Carreras F, Pons-Lladó G, Martí-Vilalta JL (2006). Almost perfect concordance between simultaneous transcranial Doppler and transesophageal echocardiography in the quantification of right-to-left shunts. J Neuroimaging.

[B11] Willcox TW, Mitchell SJ, Gorman DF (1999). Venous air in the bypass circuit: a source of arterial line emboli exacerbated by vacuum-assisted drainage. Ann Thorac Surg.

[B12] Maselli D, Pizio R, Musumeci F (2006). Multifrequency transcranial Doppler for intraoperative automatic detection and characterisation of cerebral microemboli during port-access mitral valve surgery. Interact Cardiovasc Thorac Surg.

